# New ZnO@Cardanol Porphyrin Composite Nanomaterials with Enhanced Photocatalytic Capability under Solar Light Irradiation

**DOI:** 10.3390/ma10101114

**Published:** 2017-09-21

**Authors:** Viviane Gomes Pereira Ribeiro, Ana Maria Pereira Marcelo, Kássia Teixeira da Silva, Fernando Luiz Firmino da Silva, João Paulo Ferreira Mota, João Paulo Costa do Nascimento, Antonio Sérgio Bezerra Sombra, Claudenilson da Silva Clemente, Giuseppe Mele, Luigi Carbone, Selma Elaine Mazzetto

**Affiliations:** 1 Laboratório de Produtos e Tecnologia em Processos (LPT), Departamento de Química Orgânica e Inorgânica, Universidade Federal do Ceará, 60440-900 Fortaleza, Brazil; vivianegpribeiro@live.com (V.G.P.R.); aninhamarcelo@yahoo.com.br (A.M.P.M.); kassiateixeira.kts@gmail.com (K.T.d.S.); fernandin@live.com (F.L.F.d.S.); jpfmpro@gmail.com (J.P.F.M.); claudenilsonsc@gmail.com (C.d.S.C.); selma@ufc.br (S.E.M.); 2 Laboratório de Telecomunicações e Ciências e Engenharia de Materiais (LOCEM), Departamento de Física, Universidade Federal do Ceará, 60440-970 Fortaleza, Brazil; jpquimico2@hotmail.com (J.P.C.d.N.); asbsombra@gmail.com (A.S.B.S.); 3 Dipartimento di Ingegneria dell’Innovazione, Università del Salento, Via Arnesano, 73100 Lecce, Italy; 4 CNR NANOTEC—Istituto di Nanotecnologia c/o Campus Ecotekne, Università del Salento, Via Monteroni, 73100 Lecce, Italy; luigi.carbone@nanotec.cnr.it

**Keywords:** photocatalysis, ZnO, CNSL, porphyrins, rhodamine B, sunlight irradiation

## Abstract

This work describes the synthesis, characterization, and photocatalytic activity of new composite nanomaterials based on ZnO nanostructures impregnated by lipophlilic porphyrins derived from cashew nut shell liquid (CNSL). The obtained nanomaterials were characterized by X-ray diffraction (XRD), UV-Vis diffuse reflectance spectroscopy (DRS), Fourier transform infrared spectroscopy (FT-IR), transmission electron microscopy (TEM), and steady-state photoluminescence spectra (PL). The results confirm nanostructures showing average diameter of 55 nm and an improved absorption in the visible region. Further, the FTIR analysis proved the existence of non-covalent interactions between the porphyrin molecules and ZnO. The photocatalytic activity of prepared photocatalysts was investigated by degradation of rhodamine B (RhB) in aqueous solution under visible light irradiation and natural sunlight. It was demonstrated that the photocatalytic activity increases in the presence of the porphyrins and, also, depends on the irradiation source. The development of composite photocatalysts based on porphyrins derived from CNSL provides an alternative approach to eliminate efficiently toxic wastes from water under ambient conditions.

## 1. Introduction

There is growing concern about water pollution due to increased industrial activity worldwide. Among the main pollutants present in wastewaters, dyes are one of the most compromising of the aquatic life and potable water quality. Every day, large amounts of dyes are discharged into the water, from textile, paper, plastic, and cosmetics industries [1,2,3]. Many of them are of a synthetic origin with complex aromatic structures and difficult to degrade [4]. Conventional methods are not efficient for eliminating toxic organic dyes and, nowadays, heterogeneous photocatalysis using nanostructured semiconductors has been regarded as a promising technology and applied to the purification of contaminated water [2,5,6]. Photocatalytic technology has potential economic and significant environmental benefits because it has advantages, such as being non-toxic, inexpensive, highly efficient, and reusable [7]. The process is based on the generation of the electron-hole pairs on the catalyst surface that induce the production of highly-reactive radicals in solution [7,8].

Among the semiconductors, zinc oxide (ZnO) has been be shown to be an excellent photocatalyst due to its benefits and favorable attributes, such as a wide band gap (e.g., ~3.2 eV), good optoelectronic properties, high quantum efficiency, mechanical-thermal stability, non-toxicity, and versatility in synthesis [9,10,11]. Furthermore, at the nanoscale, ZnO has demonstrated improved photocatalytic performance due to the enhancement of the surface area and the quantum confinement effect [12,13,14]. However, the main disadvantage of ZnO lies in the fact that it is active only under UV light irradiation, due to its large band-gap, thus limiting its photocatalytic applications, especially under sunlight.

Without a doubt, sunlight is by far the source with the highest energy potential in the world [15] and the search for photocatalysts that make use of this clean and abundant energy, besides being appropriate, represents a sustainable alternative. There is about 47% visible light (400 < λ < 700 nm) in the solar spectrum which can, and should, be used in the treatment of dyes and organic pollutants [16]. Therefore, in order to activate ZnO with visible light, many methods have been applied, such as the coupling with other semiconductors, doping with non-metals (C, N, S, or P), noble-metals, transition metal and use of photosensitizers [9,10,14,15,17,18]. Among them, the use of porphyrins as photosensitizer agents of semiconductors has been considered one the most promising methods to extend the light absorption of ZnO due their strong absorption in the visible region of the solar spectrum, good chemical stability, and higher efficiency of electron transfer, non-toxicity, and compatibility with the environmental [17,18,19]. Composites based on porphyrin immobilization on the surface of semiconductors have been considered as efficient materials in light-harvesting systems and photocatalysis [18,19,20].

Porphyrins are versatile molecules with optical, photophysical, and electrochemical properties adjustable by peripheral substitution or metallic complexation [21]. Many efforts have been made towards the development of novel porphyrins to enhance electron transfer efficiency to semicondutors, among which molecules containing mainly carboxyl, sulfonyl, or hydroxyl groups are included [22,23,24]. Moreover, although some studies using porphyrins as photosensitizers of ZnO have been performed [8,17,18,19,25,26,27], the influence of long alkyl groups in porphyrins on photocatalytic activity of these materials it still is exploited very little. In particular, in order to make the process even more environmentally friendly, it is interesting to use porphyrins derived from natural and renewable sources, such as the cardanol present in the cashew nut shell liquid (CNSL), obtained as a byproduct of agribusiness of the cashew nut (*Anacardium occidentale* L.) [28,29]. Cardanol is the main constituent of the technical CNSL (65%) and presents unique structural features that allow for application in the preparation of a variety of fine chemicals and hybrid materials [30,31,32,33].

Based on these considerations, here we report the synthesis, characterization, and photocatalytic activity of new environmentally friendly composite nanomaterials based on ZnO@porphyrin derived from cardanol. The photocatalytic performance of the composite nanomaterials was evaluated by the degradation of Rhodamine B (RhB) dye in aqueous solution under visible light irradiation and natural sunlight.

## 2. Materials and Methods

### 2.1. Materials

All the reagents and solvents used in this work were supplied by Sigma-Aldrich (St. Louis, MO, USA) and Vetec Química (Duque de Caxias, Brazil). The solvents used were methanol, ethanol, hexane, dichloromethane, dimethylformamide (DMF), and chloroform. The chemical reagents were dibromoethane, 4-hydroxybenzaldehyde, boron trifluoride diethyl etherate (BF_3_·OEt_2_), 2,3-dichloro-5,6-dicyano-p-benzoquinone (DDQ), pyrrole, copper acetate (Cu(OAc)_2_·nH_2_O), zinc oxide (ZnO), and rhodamine B (RhB). The 3-n-pentadecylphenol (hydrogenated cardanol) was obtained through chromatographic separation of the constituents of CNSL according to the established procedure reported in [34].

### 2.2. Synthesis of the Porphyrins H_2_Pp (3) and CuPp (4) from Cardanol

**Figure 1:**
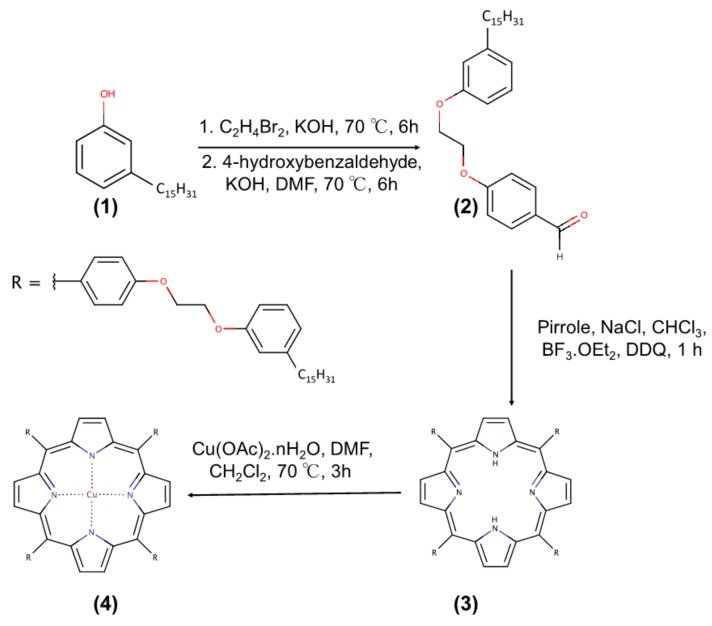
Synthetic scheme for porphyrins from cardanol: (**1**) hydrogenated cardanol; (**2**) aldehyded precursor; (**3**) H_2_Pp; (**4**) CuPp.

Figure 1 shows the steps and reagents used in synthetic route of porphyrins obtained from hydrogenated cardanol. The compound 4-[2-(3-n-pendacylphenoxy)-ethoxy]benzaldehyde (**2**), derivated from hydrogenated cardanol (**1**), was prepared and characterized according to the procedure described by Mota et al. [28]. The procedure followed two steps. The first step involved the synthesis of the brominated precursor (1-(2-bromoethoxy)-3-pentadecylbenzene) which was obtained by reaction of hydrogenated cardanol (13.16 mmol) and 1,2-dibromoethane (174.00 mmol) using KOH (39.49 mmol) as the base. The system was maintained at 70 °C, stirring for about 6 h. The product was purified by recrystallization according to the following procedure: 50 mL of distilled water was added to the reaction mixture and the solution was filtered. After this, 100 mL of methanol was added to the obtained solid and the solution was heated at 65 °C for 10 min, yielding two phases: one colorless and the other yellow. The colorless phase was separated and the solution cooled for 20 min at −10 °C to recrystallize the product of interest. Then, the product was filtered and a white solid was thus obtained with a reaction yield of 86% (4.6 g). In the second step, the brominated precursor (7.30 mmol) and 4-hydroxybenzaldehyde (10.90 mmol) were used and accordingly KOH (23.30 mmol) as a base in 50 mL of DMF was added. The system was stirred at 100 °C for 6 h, then purified by recrystallization following the same above reported procedure. The product was obtained as a white solid with 56% (1.8 g) of reaction yield. 

The compound 5,10,15,20-*tetra*-[4-(2-(3-pentadecyl)phenoxy)ethoxy]phenylporphyrin (**3**) was synthesized according to the procedure reported in the literature [35]. It was obtained by reacting the compound **2** (2.21 mmol) with pyrrole (2.21 mmol) and NaCl (55.25 mmol). The mixture was dissolved in 50 mL of chloroform containing 0.8% ethanol and kept under stirring for 10 min under inert atmosphere (N_2_). Then, BF_3_·OEt_2_ (0.72 mmol) was added. After about 10 min, DDQ (1.64 mmol) was added to the mixture. The system remained under stirring at room temperature and in an inert atmosphere for 1 h. At the end of the reaction, the product was concentrated and treated with 100 mL of a mixture DMF/ethanol (8:2 V/V) under vigorous stirring. The crude product was filtered and then purified by chromatography on a silica gel column with a dichloromethane as eluent. The product was obtained as a purple solid with a 27% (300 mg) of reaction yield.

**H_2_Pp:** Yield 27%. Anal. Calcd. for C_136_H_182_N_4_O_8_: C, 81.64%; H, 9.17%; N, 2.80%. Found: C, 81.14%; H, 9.76%; N, 2.99%. MALDI-TOF MS *m*/*z*: 2000 [M]^+^; Molecular weight: 2001 amu; ^1^H-NMR (CDCl_3_, 300 MHz): δ, ppm: −2.70 (s, 2H, NH), 0.98 (t, 12H, CH_3_), 4.59 (dd, 16H, O–(CH2)_2_–O), 6.87–8.15 (m, 24H, *o*-Ph, *m*-Ph and *p*-Ph), 8.89 (s, 8H, *β*-Pyr); FT-IR (KBr), cm^−1^: 966 (δ N–H), 1286 (ν C–N), 2848 and 2918 (ν C–H Aliphatic), 3034 and 3068 (ν C–H Aromatic), 3325 (ν N–H); UV-Vis, CHCl_3_ (λ_max_, nm): 421; 519; 556; 594; 651.

The compound Cu (II) 5,10,15,20-*tetra*-[4-(2-(3-pentadecyl)phenoxy)ethoxy]phenyl porphyrin (**4**) was synthesized according to the procedure reported by [29], with some modifications. Initially, the compound **3** (0.05 mmol) was solubilized in 20 mL of dichloromethane and the solution heated to 70 °C. Then, Cu(OAc)_2_·nH_2_O (5.00 mmol) was dissolved in 20 mL of DMF. The system was stirred under nitrogen for approximately 3 h. Then, the reaction mixture was treated in a separatory funnel containing 40 mL of distilled water and 40 mL of dichloromethane. The organic phase was separated, concentrated, and purified by chromatography using a silica gel column and dichloromethane/hexane (7:3, V/V) as eluent. The product was obtained as a pink solid with a 93% (95 mg) reaction yield.

**CuPp:** Yield 93%. Anal. Calcd. for C_136_H_180_N_4_O_8_Cu: C, 79.20; H, 8.80; N, 2.72. Found: C, 79.28; H, 9.75; N, 2.16%. MALDI-TOF MS *m*/*z*: 2062 [M]^+^; Molecular weight: 2062 amu; ^1^H-NMR (CDCl_3_, 300 MHz): δ, ppm: 0.88 (brs, 12H, CH_3_), 4.46 (brs, 16H, O–(CH2)_2_–O), 6.85 (brs, *o*-Ph, *m*-Ph and *p*-Ph); FT-IR (KBr), cm^−1^: 998 (N–M), 1287 (ν C–N), 2849 and 2917 (ν C–H Aliphatic), 3029 and 3067 (ν C–H Aromatic); UV-Vis, CHCl_3_ (λ_max_, nm): 419; 541; 578.

### 2.3. Preparation of the H_2_Pp-ZnO and CuPp–ZnO Photocatalysts

The photocatalysts were obtained according to the method previously reported by [17], with some modifications. Initially, a 4 µmol amount of the porphyrin H_2_Pp was dissolved in 15 mL of dichloromethane and then 1 g of nanoparticulate ZnO was added into this solution. The mixture was placed under ultrasound irradiation at 40 °C for 1 h and, afterwards, stirred at room temperature for 24 h. Then, the solvent was removed under vacuum and the H_2_Pp–ZnO photocatalyst was collected. The same procedure was performed with CuPp to obtain nanostructures of CuPp–ZnO.

In particular, the samples impregnated with of 4 µmol per gram of ZnO exhibited the highest photoactivity. For this reason this amount was considered as the optimal loading of porphyrins onto the surface of the ZnO powders. Increasing the loading (5 and 6 µmol of Pps/1 g ZnO) no further beneficial effect were observed. A decrease of photoactivity was observed for loading ranges between 3 µmol Pps/1 g ZnO and bare ZnO. 

### 2.4. Sample Characterizations

The X-ray diffraction (XRD) patterns were recorded on X-ray powder diffractometer Xpert Pro MPD (Panalytical, Almelo, Netherlands) using CoKα radiation (λ = 1.7889 Å) operated at 40 kV and 30 mA over the 2θ range from 20° to 80°. The phase identification analysis was made by comparing the obtained powder diffractograms with standard patterns from Inorganic Crystal Structure Database (ICSD). UV-Vis diffuse reflectance spectra (DRS) were recorded on a Shimadzu UV-3100 spectrophotometer (Kyoto, Japan) by using BaSO_4_ as a reference. FT-IR spectra were recorded on a Perkin Elmer Frontier spectrometer (Boston, MA, USA) using KBr pellets. The morphologies of the samples were examined by transmission electron microscopy (TEM) using a JEOL JEM-1011 instrument (Tokyo, Japan), operating at 100 kV and equipped with a CCD camera ORIUS 831 from Gatan (Pleasanton, CA, USA). Photoluminescence spectra (PL) were measured on a high-resolution Ocean Optics HR4000 CG-UV-NIR spectrometer (Winter Park, CO, USA), using a multi-channel LED light source (MCLS) as the source of excitation at a wavelength of 385 nm and a maximum output power of 9 mW.

### 2.5. Photocatalytic Activity Measurements

**Figure 2:**
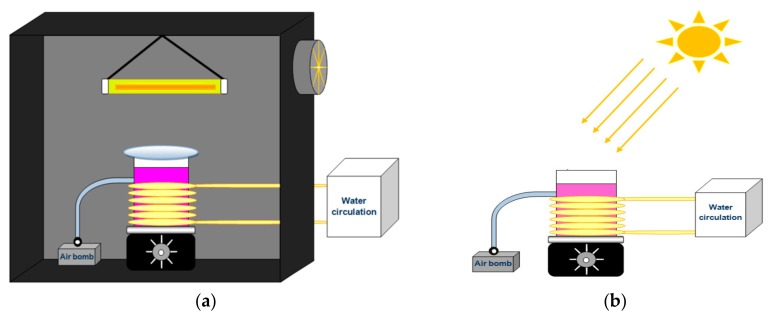
Photocatalytic reactor under 300 W halogen lamp irradiation (**a**) and under natural solar irradiation (**b**).

The photocatalytic activity of H_2_Pp–ZnO and CuPp–ZnO samples were measured by monitoring the degradation of RhB under visible light and natural sunlight irradiation. For measurements under visible light irradiation, a 300 W halogen lamp, with a wavelength range of 380–780 nm, was used as a light source and a 420 nm cutoff filter was placed between the lamp and the beaker to absorb the UV light. The distance between the lamp and the solution was set at 12 cm and an average light intensity of 208 W/m^2^ was measured at the position of the sample surface, through an HD 2302-0 (Delta OHM, Padua, Italy) radiometer. The reactor atmosphere was thermostated at 25 °C by means of continuous water circulation. The entire experimental setup was then located inside a black box, as shown in Figure 2a. In each experiment, 50 mg of photocatalyst and 50 mL of RhB solution (1 × 10^−5^ mol/L) were employed, while stirring and with constant bubbling of air. Prior to the photocatalytic performance test, an adsorption equilibrium was performed in the dark to determine the time to reach the adsorption equilibrium, in which the time of 30 min was found. Thus, before light irradiation, the solution was stirred for 30 min to establish the adsorption–desorption equilibrium of dye onto photocatalyst. Then, during the irradiation, about 3 mL of solution was periodically collected and analyzed by a UV-Vis spectrophotometer (Cary 60, Agilent, Santa Clara, CA, USA) at a wavelength of 554 nm.

Concerning investigations under sunlight irradiation, the experimental setup was arranged in an external environment (Figure 2b) during consecutive sunny days in August 2016 between 10:30 a.m. and 2:00 p.m. (GPS coordinates: 3°44′45.5′′ S, 38°34′37.7′′ W). The average light intensity at the position of the sample surface was about 530 W/m^2^. The same experimental protocol previously reported was conducted under these conditions as well.

The reuse tests of the photocatalyst was carried out following the same procedure of photodegradation of RhB. For the first cycle, similar parallel tests were executed five times. In each test, the catalyst was collected by centrifugation, washed with ethanol and dried at room temperature overnight. After this, it was reused in a new photocatalytic cycle. The photostability was analyzed by DRS spectra after the last cycle.

## 3. Results and Discussion

### 3.1. XRD Analysis, FT-IR Spectroscopy, UV-Vis DRS and Photoluminescent Properties

**Figure 3:**
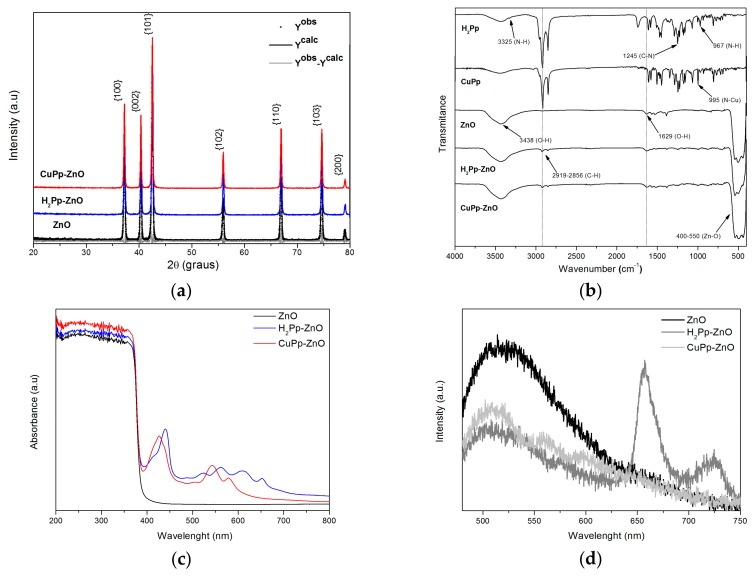
(**a**) XRD patterns of the photocatalysts; (**b**) FT-IR spectra; (**c**) UV-Vis reflectance spectra; and (**d**) photoluminescence spectra.

The XRD patterns of the bare ZnO and of H_2_Pp–ZnO and CuPp–ZnO synthesized photocatalysts are shown in Figure 3a. As can be observed, the ZnO sample showed well-defined diffraction peaks at {100}, {002}, {101}, {102}, {110}, {103}, and {200} corresponding to the hexagonal wurtzite crystal structure [36], with space group P63mc, as confirmed by Rietveld refinement (ICSD 65119). In addition, it is largely evident that the patterns of H_2_Pp–ZnO and CuPp–ZnO fit the same profile as observed for pure ZnO, whereby no diffraction peaks were detected related to the porphyrins due to its low doping amount [26].

The FT-IR spectra of H_2_Pp, CuPp, ZnO, H_2_Pp–ZnO, and CuPp–ZnO are shown in Figure 3b. The H_2_Pp shows axial and angular deformation of the N-H bond of the macrocycle at 3327 cm^−1^ and 967 cm^−1^ respectively, two bands between 2919 and 2856 cm^−1^ attributed to stretching of the C–H bond of the aliphatic side chains derived from cardanol and a band at 1245 cm^−1^ due to the axial deformation of the C–N bond typical of the pyrrole ring [37]. For metalloporphyrin CuPp, the main change observed is in the disappearance of N–H vibrations and the appearance of N–Cu absorption band at 995 cm^−1^ [38]. Furthermore, in the spectra of ZnO, the observed characteristic peaks corresponding to the absorption bond of Zn–O and Zn–O–Zn in the range of 400–500 cm^−1^. The peak appearing at 3438 cm^−1^ is related to the OH groups and water adsorbed onto ZnO [8,39]. Additionally, concerning the H_2_Pp–ZnO and CuPp–ZnO photocatalysts, it is to be noted that the ZnO-related bands were not displaced, and the characteristic peaks of porphyrinic chains could be detected at very low intensity. This can be interpreted as a weak interaction between ZnO and H_2_Pp or CuPp, that is, interactions of a non-covalent nature, such as Van der Waals or hydrogen bonding [40].

Figure 3c shows the UV-Vis diffuse reflectance spectra of the bare ZnO and composite nanomaterials recorded in the range of 200–800 nm. Pure ZnO shows a strong absorption in the UV region with a limit around λ = 385 nm, corresponding to the oxide band gap [17], and no considerable absorption in the visible region of the spectrum. On the other hand, the photocatalysts presented strong absorption bands, characteristics of H_2_Pp and CuPp porphyrins, in the visible region. Their respective UV-Vis spectra can be seen in Figure S1 of the Electronic Supplementary Information (ESI). Typical UV-Vis spectra of porphyrins exhibit absorptions in two regions respectively known as the Soret or B-band (~380–420 nm) and the Q-bands (~500–800 nm) which are attributed to π–π* transitions. The metalloporphyrin CuPp shows a blue shift in the soret-range compared to free-base porphyrin H_2_Pp because of the atomic orbitals of metallic center strongly overlapping with the occupied molecular orbitals of the ligand and, thus, resulting in a increase of the electronic transition energy [41]. Moreover, a red-shift was observed studying the DRS of the composite nanomaterials when they were compared with the data obtained for the porphyrins solution and in powder form (see Table S1 ESI), and it was more pronounced for H_2_Pp (19 nm) than CuPp (8 nm). This behavior could be related to the formation of aggregates of porphyrins of J-type on catalyst regarding monomeric form of the porphyrins in solution, while the peak at 396 nm indicate the presence of H-type in powder form. Nevertheless, J-aggregate formation does not represent a limitation for photocatalytic applications since the directional energy transfer makes the aggregate more suitable for broader spectral sensitization, besides improving the interfacial charge separation on the ZnO surface [20]. This result clearly indicates that the adsorption of the cardanol-derived porphyrins onto the surface of the oxide nanostructures enhances and extends the range of light absorption in the visible part of solar spectrum.

Additionally, the photoluminescent properties of ZnO, H_2_Pp–ZnO, and CuPp–ZnO photocatalysts were studied, as shown in Figure 3d. Photoluminescent spectra of semiconductor oxides originates from the radiative recombination of photogenerated electron–hole pairs [11]. After excitation of the ZnO near the energy band edge (λ = 385 nm), an emission band at 521 nm, which can be ascribed to the oxygen vacancies or defects happening at the ZnO surface was observed [36,42,43]. On the contrary, the intensity of the 521 nm emission band of the H_2_Pp–ZnO and CuPp–ZnO photocatalysts, was lower than that of bare ZnO. This observation clearly indicates that an electronic interaction between porphyrin molecules and ZnO may have arisen. Generally, the weaker the emission spectrum of semiconductor materials, the higher the rate of separation of photoinduced charge carriers and, possibly, the higher the photocatalytic activity [44]. Moreover, it is worth noting that the emission spectrum of the sample H_2_Pp–ZnO presented two intense peaks, respectively at 657 nm and 723 nm, attributed to the emission of pure porphyrin H_2_Pp [35]. The photoluminescent spectra of H_2_Pp and H_2_Pp–ZnO with visible light excitation can be seen in Figure S2 of the Electronic Supplementary Information (ESI), showing that even in the presence of ZnO, H_2_Pp fluorescence was not suppressed. Free base porphyrins generally have higher fluorescence quantum yields than metalloporphyrins being, for this reason, more efficient in the formation of singlet oxygen, a reactive species of great interest in photochemical reactions [45].

### 3.2. Morphology Analysis Based on TEM

**Figure 4:**
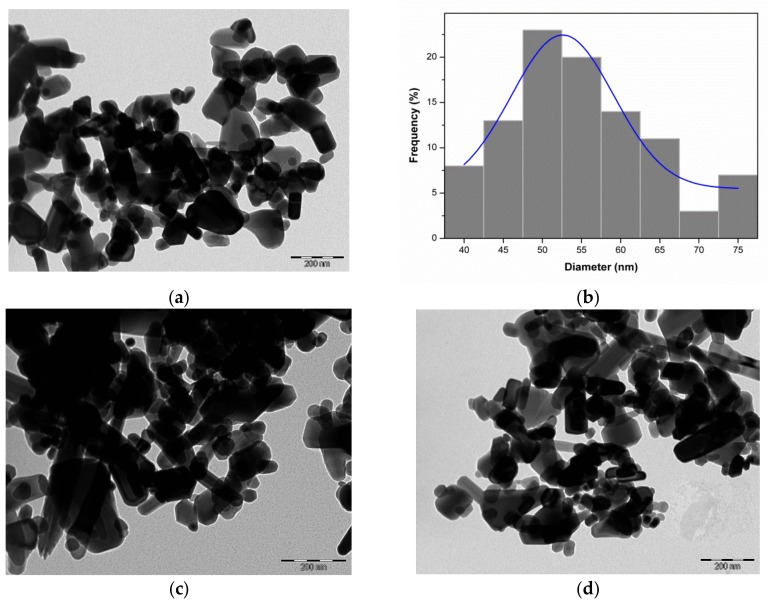
TEM image of bare ZnO (**a**) and sample size distribution (**b**); and TEM images of H_2_Pp–ZnO (**c**) and CuPp–ZnO (**d**).

With the aim at observing the size and the morphology of the photocatalysts, TEM analysis of the samples were performed. Reasonably, reports in the literature [10,14] have shown that the size and the morphology directly affect the photocatalytic activity of nanosized ZnO. Figure 4a shows TEM images of ZnO nanostructures organized as irregular agglomerates of nanoflakes and nanorods, which are typical morphologies of ZnO [10]. The diameter of ZnO nanoflakes were about 53 nm (Figure 4b), whereas the nanorods showed an average diameter of 55 nm. No changes in the original morphology could be discriminated after impregnation of the porphyrins onto the surface of the nanostructured ZnO (see Figure 4c,d). The nanometric size of the photocatalysts can be considered beneficial to photocatalysis process, since it affect the dynamics of the recombination of electron–hole pairs and enables a rapid migration of the photogenerated charges to the catalyst surface [46].

### 3.3. Effect of Catalyst Loading

**Figure 5:**
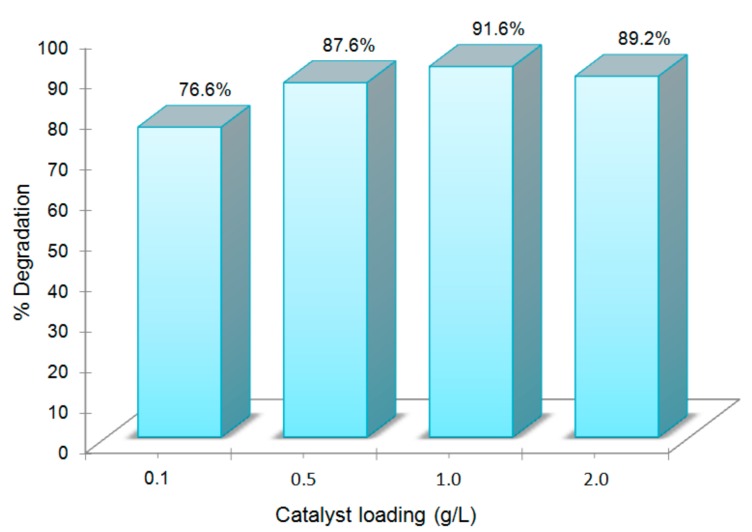
Effect of different amount of H_2_Pp–ZnO on photodegradation efficiency of RhB under visible light irradiation.

The response of the catalysts concentration on the efficiency of photodegradation in aqueous solution was investigated, as the use of a dosage above or below an optimal concentration may negatively influence the removal process. Figure 5 illustrates that the degradation rate of RhB increases with an increase in the catalyst loading, up to an amount of 1.0 g/L and decreased for an amount of 2.0 g/L. This is due to the fact that, at low concentrations there are few active sites available to catalyze the reaction, whereas, for very high concentrations, the light penetration is impaired by the excess particles in the solution, thus preventing the photons from reaching the surface of the photocatalyst and, consequently, decreasing the efficiency of the process [47]. Experiments provided 1.0 g/L as the most favorable concentration.

### 3.4. Evaluation of Photocatalytic Activity

**Figure 6:**
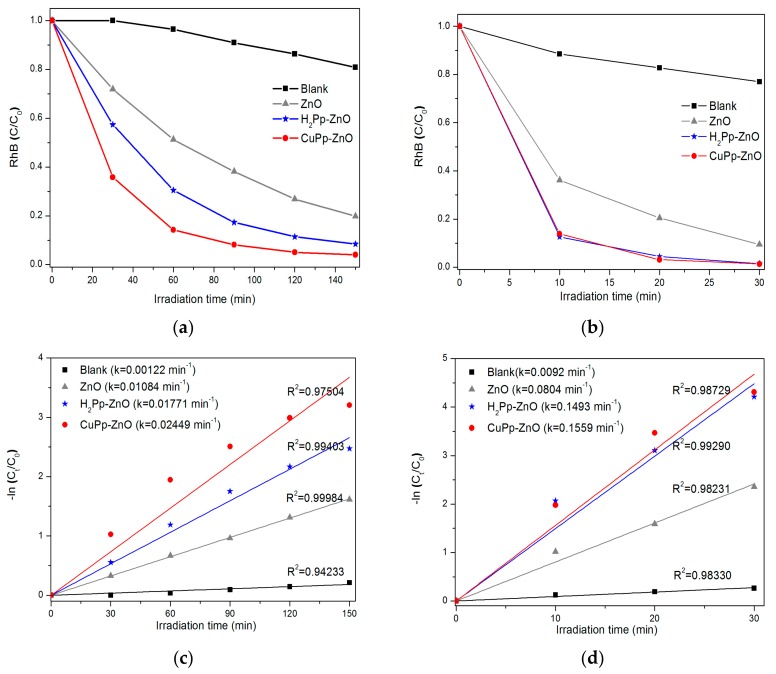
Photodegradation of RhB vs. irradiation time by bare ZnO, H_2_Pp–ZnO, and CuPp–ZnO photocatalysts (**a**) under visible light irradiation; and (**b**) under natural sunlight irradiation. Kinetic model of pseudo-first order for RhB photodegradation under irradiation of visible light (**c**); and natural sunlight (**d**).

The photocatalytic activities of ZnO, H_2_Pp–ZnO, and CuPp–ZnO were evaluated by the degradation of rhodamine B (RhB) in water under visible light and natural sunlight irradiation. Figure 6a shows the concentration variation of the dye as a function of time achieved by the decrement of the characteristic absorbance of RhB at 554 nm. The degradation profiles have been provided in Figures S3–S6 of the Electronic Supplementary Information (ESI). In the blank experiment, namely under irradiation of continuous visible light, however, in the absence of any photocatalyst, the RhB solution exhibited 19% of degradiation after 150 min. The bare ZnO showed RhB decomposition as large as 80%. In fact, ZnO does not absorb visible light due to its high band gap energy, hence, the photoactivity presented by the sample is due to the photosensitization mechanism promoted by RhB and leading to its self-oxidation on the surface of the oxide [48]. H_2_Pp-ZnO and CuPp–ZnO photocatalysts proved to be more efficient than pure ZnO, because of a percentage of RhB decomposition of 91.5% and 96.2%, respectively. It is well-known that copper porphyrins improve the separation of photogenerated charges (electron–hole pairs) by capture of electrons and, hence, increases the efficiency in photocatalytic degradation [22,49,50]. Based on these results, it is possible to affirm that the photocatalysts synthesized with the porphyrins derived from the CNSL exhibited higher activity to degrade a dye under visible light illumination compared to previous reports [17], wherein only 63% RhB photodecomposition was shown. In this direction a pivotal role is performed by the peripheral substituent of porphyrins which, because of their length and flexibility (from cardanol), contribute to improve the charge transfer to ZnO [51].

Since a majority of the solar spectrum is visible light, it is imperative to seek a way to make use of all this available solar energy, mainly in tropical countries, like Brazil. Thus, with the aim at evaluating the influence of the UV component on the photocatalytic activity of the samples, as well as the effect of visible light intensity, tests were carried out under irradiation of natural sunlight. As illustrated in Figure 6b, only 30 min irradiation of sunlight was sufficient to both porphyrin-based photocatalysts to achieve almost complete decomposition (98%) of RhB. The degradation profiles have been provided in Figures S7–S10 of the Electronic Supplementary Information (ESI). This indicates that the presence of porphyrins is crucial for better photoactivity under sunlight.

The observed increase in the rate of decomposition herein can be explained by the cooperative mechanism of process activation, respectively involving the visible light which sensitizes the porphyrins and the UV component which activates the ZnO nanoparticles. Taking into account that under visible light irradiation only the porphyrins, but not ZnO, can be activated to the excited state [17]; nevertheless, the effect of the incident light intensity on the solution which, in the first trial had a mean value of 208 W/m^2^, and under natural sunlight of 530 W/m^2^, cannot be ruled out. Some previous studies have reported that the photocatalytic efficiency increases when a higher visible light intensity is applied [46,52]. The light intensity is related to the number of photons generated, while the energy of a photon is related to its wavelength [53]. Therefore, herein we have investigated whether, upon increasing the light intensity, more radiation reaches the surface of the photocatalyst in this fashion, producing more reactive radicals in the reaction environment.

The kinetic analysis of the majority of the photocatalytic processes is based on the Langmuir–Hinshelwood model [50]. This kinetic model describes a linear relationship obtained between the reaction rate and the concentration of the substrate in the solution. The equation predicts a pseudo-first order kinetic, which is based on the assumption that the substrate is adsorbed by a photocatalyst obeying to a Langmuir isotherm, i.e., adsorption as a mono-layer and in a condition of adsorption-desorption equilibrium maintained during the process [1,54]. In other words, the adsorption-rate onto the substrate is much faster than the reaction-rate with the photogenerated charges (e^−^/h^+^). In a simplified way, the equation is described by: ln(C_0_/C) = −kt , where C_0_ and C are the concentrations of RhB in the reaction solution at irradiation time zero and t, respectively, and k (min^−1^) represents the pseudo-first-order rate constant.

Figure 6c,d shows the linearized experimental data, through which it was possible to obtain the kinetic constants for the photocatalysts under irradiation with visible light and sunlight. In general, a good linear correlation of the experimental data (R^2^ > 0.95) is observed, adjusted to the kinetic model of pseudo-first order. By evaluation of the kinetic constants (k) obtained, it can be inferred that CuPp–ZnO represents the most efficient photocatalyst under the experimental conditions investigated herein. Precisely, the rate constant of CuPp–ZnO under sunlight irradiation (k = 0.1559 min^−1^) was approximately 6.36 times greater than the value found under visible light irradiation (k = 0.02449 min^−1^). Notably, this proves that besides the presence of the UV component in solar radiation, the increase in the light intensity promotes a gain in the rate of photodegradation.

### 3.5. Proposed Photocatalytic Mechanism

**Figure 7:**
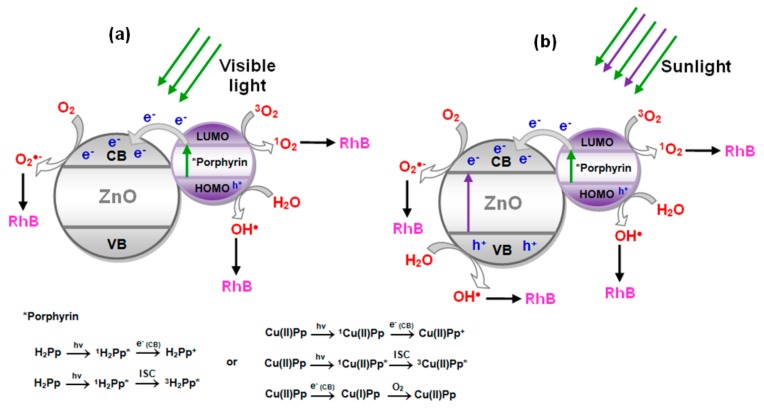
Proposed mechanism for reactive species formation by photocatalysts under visible light (**a**) and natural sunlight (**b**).

Based on the experimental results discussed above, a possible mechanism for photodegradation of RhB, which portrays the performance of H_2_Pp–ZnO and CuPp–ZnO, is proposed in Figure 7. Essentially, the photocatalytic process is based on the specific acceleration of oxidation and reduction reactions due to the presence of a light activated photocatalyst. The process is initiated when the semicondutor (ZnO), absorbs photons with energy greater than its band gap, that is, the energy between the valence band (VB) and the conduction band (CB). Thus, after the light absorption, electrons (e^−^) are transferred from the valence band to the conduction band, which leads to hole (h^+^) generation in the valence band [16]. These photogenerated electrons and holes migrate to the catalyst surface and tend to perform redox reactions with oxygen and water present in solution to produce superoxides (•O_2_^−^) and hydroxyl (•OH) radicals, respectively. These highly-reactive radicals are responsible for reactign with RhB and degrade it during the process. However, when under visible light irradiation (Figure 7a), ZnO it cannot be excited due to its large band-gap (3.2 eV), predominating the photosensitization process by the photosensitizer. In this case, first the electrons are excited from the highest occupied molecular orbital (HOMO) to the lowest unoccupied molecular orbital (LUMO) of H_2_Pp or CuPp, and are then transferred into CB of ZnO, subsequently forming •O_2_^−^ for degrade the RhB [25]. Simultaneously, holes in the sensitizer can react with water to produce hydroxyl radicals (•OH) and also act as an oxidizing agent of RhB [27,55,56]. In addition, porphyrins are able to produce singlet oxygen (^1^O_2_) [45], a reactive oxygen species formed by the interaction of sensitizer excited triplet state with molecular oxygen, contributing toward the oxidation process of RhB. In particular, is associated with fluorescence behavior of porphyrin, here demonstrated in the photoluminescent analysis of composite nanomaterials. The fact that CuPp–ZnO presents higher photocatalytic performance is due to the metal operating as an electron trap changing the oxidation state during the process and participating in the reduction of O_2_ to •O_2_^−^ [49,50]. 

The beneficial effect on the photoreactivity observed under sunlight irradiation (Figure 7b), could be ascribed to a cooperative mechanism similar to that previously reported involving a class of photocatalysts based on semiconductor@sensitizer (e.g., TiO_2_@porphyrin, TiO_2_@ *bis*-phthalocyanines) [57]. In that context, the photoexcitation of the semiconductor was essential for the improvement of the photocatalytic process that confirmed the existence of a synergic action involving both the photoexcitation of the semiconductor (UV light component activing ZnO) and the photoexcitation of the sensitizer occurring also under visible light. Due to this cooperative mechanism the enhanced production of reactive species (•OH, •O_2_^−^, etc.) generated in the system was considered responsible for the more efficient degradation of RhB. In this situation, the presence of cardanol porphyrins is very important to increase the process efficiency due to their ability to capture the visible component of sunlight, as well as to suppress the recombination of photogenerated charges [17,19]. Therefore, photocatalytic efficiency is improved in large part by the synergistic effect of cardanol porphyrins, which collect visible light, and the high performance of ZnO under UV irradiation.

### 3.6. Reusability of Photocatalyst

**Figure 8:**
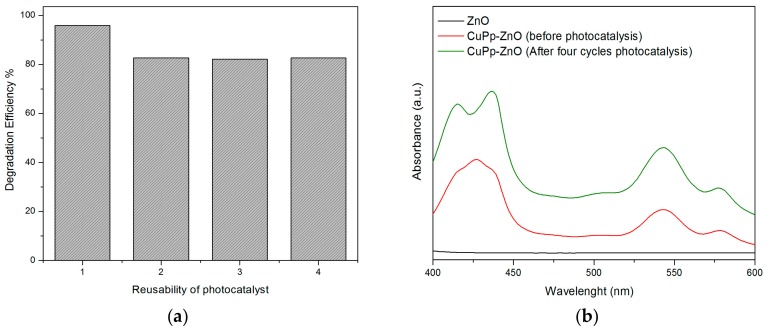
Reusability of CuPp-ZnO in the photodegradation of RhB (**a**); and UV-Vis diffuse reflectance spectra after last cycle of CuPp–ZnO photocatalyst (**b**).

It is possible to consider that porphyrins simply cannot resist the oxidative stress to which they are subjected in the photocatalytic process. Regarding this, a past study reports that the dye (RhB), itself, undergoes a series of oxidation steps which lead its degradation, reducing the porphyrin (Pp^*+^) to its ground state [17], while other authors suggest the use of sacrificial electron donors (i.e., triethanolamine) to sensitizer regeneration [27]. However, Sardar et al. [58] reported that water, itself, acts as an electron donor to regenerate the photosensitizer, replacing the need for any undesirable sacrificial electron donors. In many cases, photostability can be demonstrated by means of infrared and UV-Vis spectroscopy techniques. Thus, to prove the photostability of porphyrins and the reusability of photocatalyst, analysis of UV-Vis diffuse reflectance spectroscopy was performed, after some successive cycles of RhB photodegradation. As shown in Figure 8a, after four cycles, the degradation efficiency of CuPp–ZnO decreased from 96.2% to 82.7%, indicating a slow reduction of its photocatalytic efficiency. The reduction of the photocatalytic activity could be due to the adsorption of intermediate species of degraded RhB upon composite nanomaterial. UV-Vis diffuse reflectance spectra of CuPp–ZnO, collected after several cycles of photocatalysis (Figure 8b), showed that the Soret and Q bands of the CuPp are still present, though with minor modifications, which might be attributed to the byproducts adsorbed on photocatalyst or the formation of porphyrin H-aggregates [20]. Therefore, CuPp–ZnO has proved to be a photocatalyst which can be in an effective and efficient way employed in environmental decontamination processes.

Although many authors, concerning the mechanism of photosensitization, have indicated a peripheral polar substituent of porphyrins as responsible for a significant electronic interaction with the semiconductive support [22,27,50], thus promoting the raise of the photocatalytic performance, in the present study, it was verified that the functionalization of porphyrins with non-polar chemical groups can also act effectively in the photocatalytic processes.

## 4. Conclusions

In summary, in the present work new composite nanomaterials with enhanced photocatalytic properties were successfully achieved through impregnating the CNSL-derived porphyrins onto ZnO. The FTIR analysis showed that interaction between porphyrins and ZnO are of a non-covalent nature. The samples H_2_Pp–ZnO and CuPp–ZnO presented nanometric sizes and improved light absorption in the visible region, both beneficial properties for application in photocatalysis. The presence of porphyrins has proved to increase the recombination times of charge carriers photogenerated in the ZnO, it is confirmed by photoluminescence. The analysis of the photodegradation process of RhB promoted by sunlight, allowed us to establish the positive effect of the incident light intensity on the solution, through a cooperative mechanism involving the UV component of the incident radiation. The results demonstrate the effectiveness photocatalytic of composite nanomaterials obtained by non-covalent approach to photosensitize ZnO with porphyrins derived from renewable sources. Thus, the production of alternative photocatalysts, like these that make use of sunlight-induced photocatalysis, are extremely important to the environment and should be encouraged.

## Supplementary Materials

The following are available online at www.mdpi.com/1996-1944/10/10/1114/s1, Figure S1: UV–Vis spectra of H_2_Pp and CuPp in solution and powder.; Figure S2: Photoluminescence spectra of H_2_Pp and H_2_Pp-ZnO excited at 470 nm.; Figure S3: RhB degradation profile in blank experiment under visible light.; Figure S4: RhB degradation profile in the experiment with ZnO under visible light.; Figure S5: RhB degradation profile in the experiment with H_2_Pp-ZnO under visible light.; Figure S6: RhB degradation profile in the experiment with CuPp-ZnO under visible light.; Figure S7: RhB degradation profile in blank experiment under sunlight.; Figure S8: RhB degradation profile in the experiment with ZnO under sunlight.; Figure S9: RhB degradation profile in the experiment with H_2_Pp-ZnO under sunlight.; Figure S10: RhB degradation profile in the experiment with CuPp-ZnO under sunlight.; Table S1: UV–Vis data of H_2_Pp and CuPp.
