# Supplementary material for: New ZnO@Cardanol Porphyrin Composite Nanomaterials with Enhanced Photocatalytic Capability under Solar Light Irradiation
Source: Materials (Basel). 2017 Sep 21;10(10):1114. doi: 10.3390/ma10101114 (PMC5666920; doi:10.3390/ma10101114)
Supplement: Supplementary file 1 [file materials-10-01114-s001.pdf]

Electronic Supplementary Information

New ZnO@cardanol porphyrin composite nanomaterials with enhanced photocatalytic capability under solar light irradiation

Viviane G. P. Ribeiro [1], Ana M. P. Marcelo [1], Kássia T. da Silva [1], Fernando L. F. da Silva [1], João P. F. Mota [1], João P. C. do Nascimento [2], Antonio S. B. Sombra [2], Claudenilson S. Clemente [1], Giuseppe Mele [3], Luigi Carbone [4] and Selma E. Mazzetto[1]

[1]Laboratório de Produtos e Tecnologia em Processos (LPT), Departamento de Química Orgânica e Inorgânica, Universidade Federal do Ceará, 60440-900, Fortaleza, Brazil.
[2]Laboratório de Telecomunicações e Ciências e Engenharia de Materiais (LOCEM), Departamento de Física, Universidade Federal do Ceará, 60440-970, Fortaleza, Brazil.
[3]Dipartimento di Ingegneria dell'Innovazione, Università del Salento, Via Arnesano, 73100, Lecce, Italy.
[4]CNR NANOTEC-Istituto di Nanotecnologia c/o Campus Ecotekne Università del Salento, Via Monteroni, 73100 Lecce, Italy.

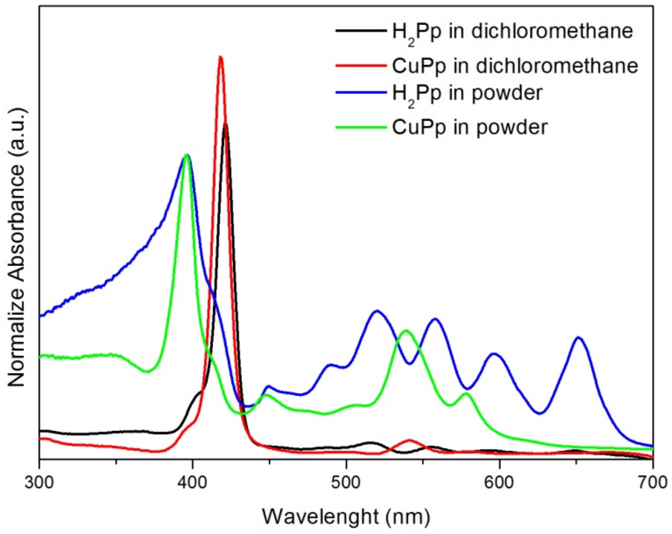

Figure S1. UV–Vis spectra of H_2Pp and CuPp in solution and powder.

Table S1. UV–Vis data of H_2Pp and CuPp.

Measure condition	Absorption λ_{max} / nm (Soret and Q bands)	
	H_2Pp	CuPp
Solution in CH_2Cl_2	421, 515, 555, 593, 649	418, 541,578
DRS in powder	396, 519, 558, 596, 651	396, 539, 578
DRS on catalyst	440, 523, 562, 609, 652	426, 543, 578

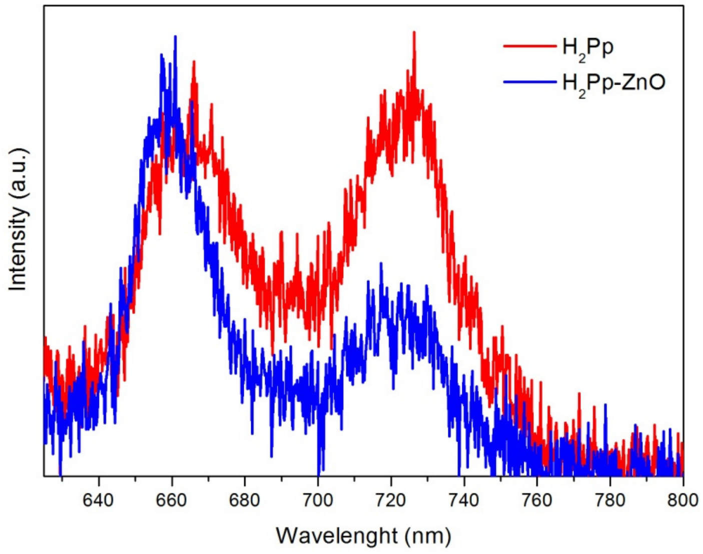

Figure S2. Photoluminescence spectra of H_2Pp and H_2Pp-ZnO excited at 470 nm.

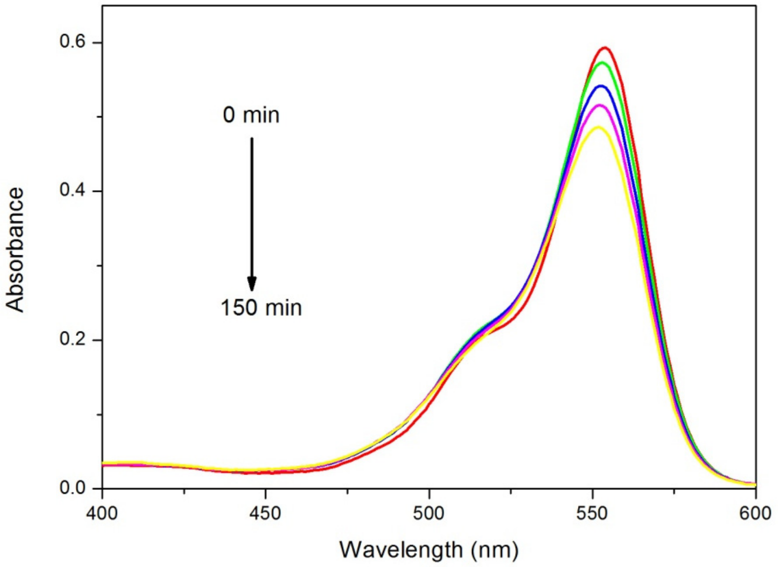

Figure S3. RhB degradation profile in blank experiment under visible light.

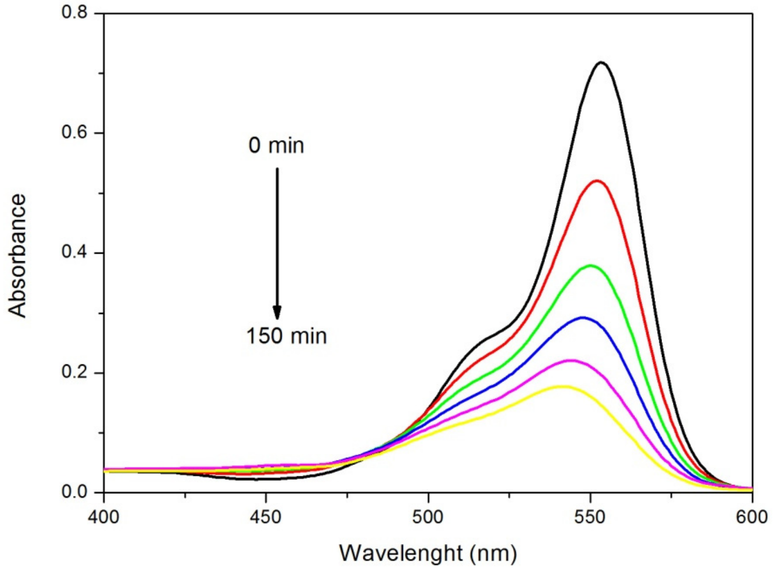

Figure S4. RhB degradation profile in the experiment with ZnO under visible light.

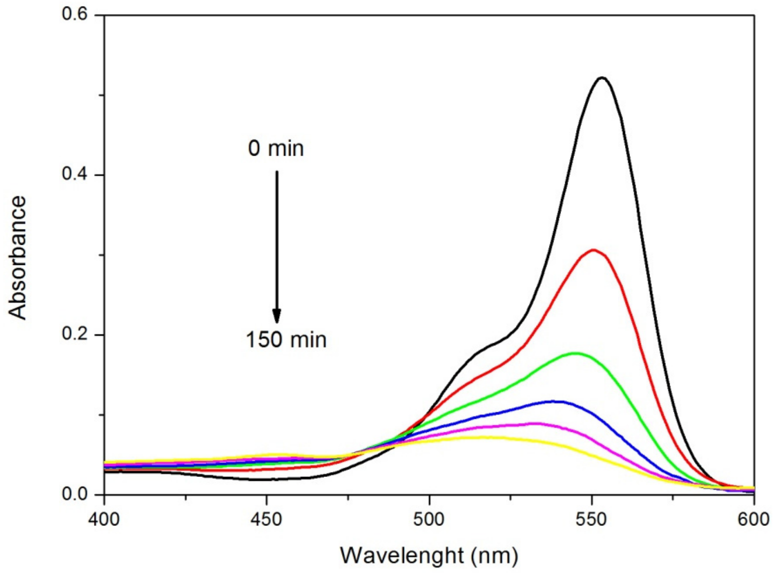

Figure S5. RhB degradation profile in the experiment with H_2Pp-ZnO under visible light.

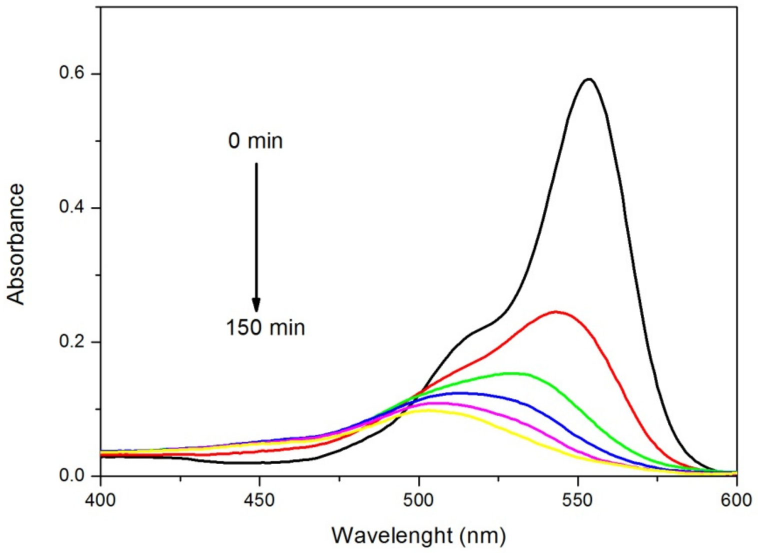

Figure S6. RhB degradation profile in the experiment with CuPp-ZnO under visible light.

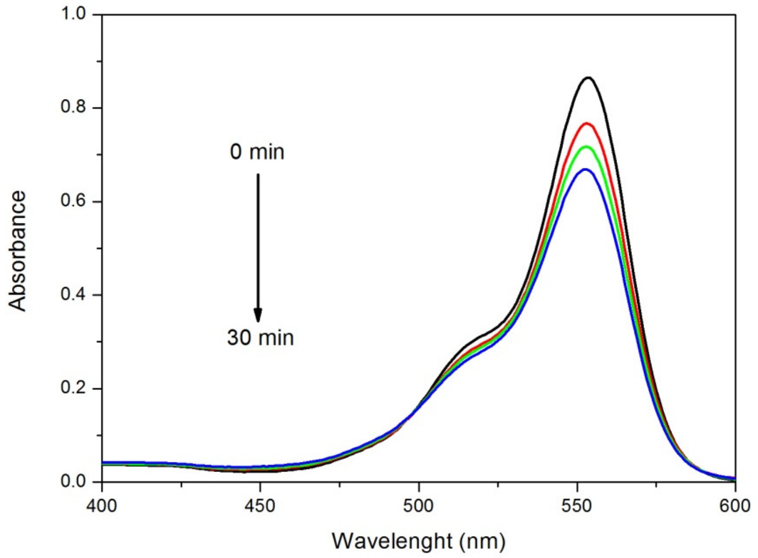

Figure S7. RhB degradation profile in blank experiment under sunlight.

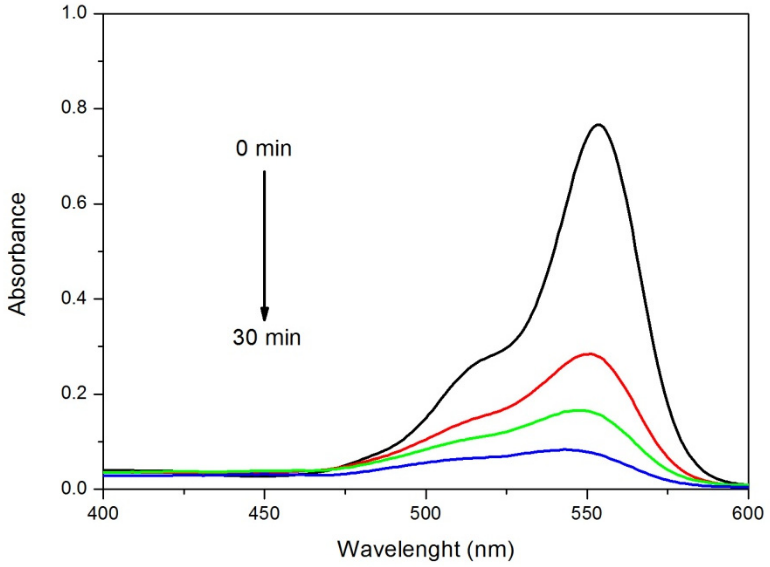

Figure S8. RhB degradation profile in the experiment with ZnO under sunlight.

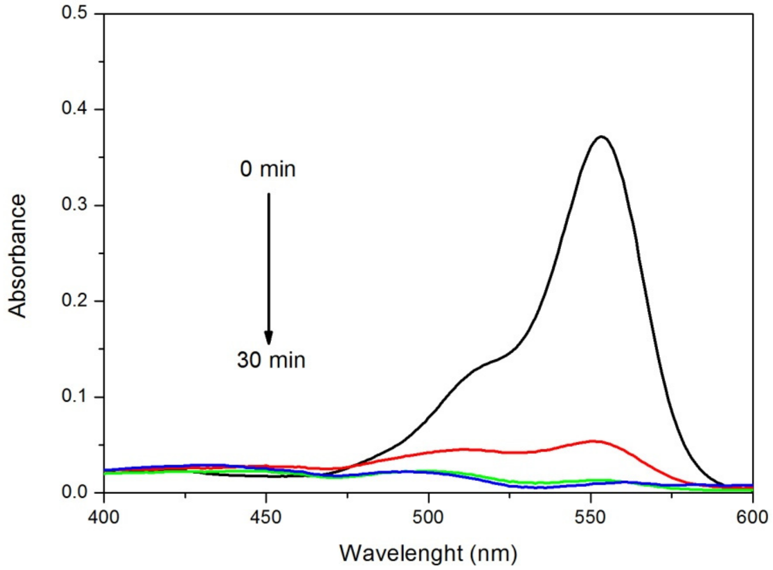

Figure S9. RhB degradation profile in the experiment with H_2Pp-ZnO under sunlight.

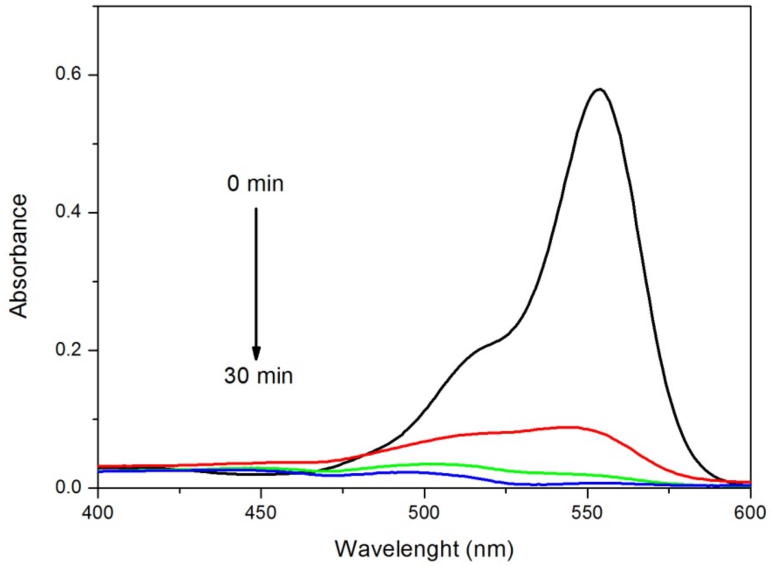

Figure S10. RhB degradation profile in the experiment with CuPp-ZnO under sunlight.
